# Specificity and context in post-exercise recovery: it is not a one-size-fits-all approach

**DOI:** 10.3389/fphys.2015.00130

**Published:** 2015-04-24

**Authors:** Geoffrey M. Minett, Joseph T. Costello

**Affiliations:** ^1^School of Exercise and Nutrition Sciences, Queensland University of TechnologyKelvin Grove, QLD, Australia; ^2^Institute of Health and Biomedical Innovation, Queensland University of TechnologyKelvin Grove, QLD, Australia; ^3^Extreme Environments Laboratory, Department of Sport and Exercise Science, University of PortsmouthPortsmouth, UK

**Keywords:** muscle damage, fatigue, cryotherapy, cold water immersion, training adaptation

The concept of specificity of exercise prescription and training is a longstanding and widely accepted foundation of the exercise sciences. Simply, the principle holds that training adaptations are achieved relative to the stimulus applied. That is, the manipulation of training variables (e.g., intensity or loading, mode, volume, and frequency) directly influences the acute training stimulus, and so the long-term adaptive response (Young et al., 2001; Bird et al., 2005). Translating this concept to practice then recommends that exercise be prescribed specific to the desired outcomes, and the more closely this is achieved, the greater the performance gain is likely to be. However, the cardiovascular and metabolic adaptations traditionally associated with long, slow distance training types, similarly achieved using high-intensity training methods (for a review see Gibala et al., 2012), highlights understanding of underlying physiology as paramount for effective training program design. Various other factors including illness, sleep, and psychology also impact on the training stimulus (Halson, 2014) and must be managed collectively with appropriate post-exercise recovery to continue performance improvements and reduce overtraining and injury risks (Kenttä and Hassmén, 1998).

Despite the emphasis that is placed on specificity in the application of the desired training stimulus, it is noteworthy that this concept receives less attention within the post-exercise recovery literature. Indeed, most recovery strategies are intended to treat only symptoms of exercise-induced muscle damage by blunting inflammatory responses associated with disturbances to the structural integrity of the exercised musculature (Minett and Duffield, 2014). Be it through lifestyle (e.g., active recovery, sleep), physiological (e.g., post-exercise cooling, massage, compression), or nutritional and pharmacological interventions (e.g., supplements, anti-inflammatory medications), these common recovery techniques aim to hasten regenerative processes below the neuromuscular junction with limited consideration for other causative mechanisms (Minett and Duffield, 2014). Compounded by the use of different exercise tasks under varying environmental conditions (e.g., hot vs. thermo-neutral temperatures) and contexts (e.g., isolated vs. repeated exercise bouts, pre-season vs. competition), this tendency for a *one-size-fits-all* approach to post-exercise recovery is likely to contribute to contrasting reports of the efficacy of many techniques. While these concerns could indicate the need for greater understanding of the mechanistic demands of specific exercise tasks and post-exercise recovery protocols so to be suitably matched, they are of great consequence in applied settings where maladaptation may be the result of inappropriate recovery practices (Kenttä and Hassmén, 1998).

The research narrative surrounding post-exercise cooling for recovery reflects this point. A derivative of the use of ice as a therapeutic treatment of soft tissue injuries, the proposed benefits of acute cooling interventions on recovery after exercise relate to peripheral vasoconstriction centralizing blood volume away from exercised musculature (Bleakley and Davison, 2009). This is proposed to benefit metabolite removal, biochemical expression of damage and inflammation, swelling and soreness (Bleakley and Davison, 2009; Costello et al., 2013). While a series of meta-analyses show considerable variance in the effectiveness of post-exercise cooling in optimizing performance return (e.g., strength, jump, and sprint variables) (Bleakley et al., 2012; Leeder et al., 2012; Poppendieck et al., 2013), evidence for a disconnect between the rise in blood-based muscle damage markers and the recovery of neuromuscular force production is noteworthy (Pointon et al., 2012; Minett et al., 2014). It could be reasoned that the indirect nature of biochemical time course expressions of common variables reported during recovery (e.g., creatine kinase) may not necessarily directly reflect concurrent neuromuscular function, though it does at least question the traditional rationale for using post-exercise cooling and how it influences performance recovery. Further, such reports give strength to the argument that physiological rationale for post-exercise cooling is limited and that any ergogenic influences reflect a perceptual or placebo effect (Broatch et al., 2014).

Irrespective of whether post-exercise cooling benefits recovery through the treatment of exercise-induced muscle damage or through other means, the specificity of administration of this intervention becomes key. Most pertinent, changes in circulatory dynamics and muscle metabolism as a result of post-exercise cooling (Vaile et al., 2011; Ihsan et al., 2013) seemingly contrast the blood flow needs required for muscle protein synthesis and training adaptation to occur (Yamane et al., 2006; Fröhlich et al., 2014). Roberts et al. (2014) recently suggested that the greater work capacity achieved using cold-water immersion recovery after resistance training could facilitate advantageous chronic adaptations, with similar conclusion drawn after the maintenance of force output following intermittent-sprint performance (Pointon et al., 2012; Minett et al., 2014). Contrastingly, however, interactions between the use of post-exercise cooling recoveries and training adaptation may be exercise specific, with both positive (Halson et al., 2014) and negative findings (Fröhlich et al., 2014) reported in cyclists and strength trained individuals, respectively. For example, while highly speculative, it might be hypothesized that the upregulation of PGC-1α expression and nitric oxide production after post-exercise cooling could stimulate GLUT4 translocation and muscle glucose uptake (Ihsan et al., 2014), thus augmenting mRNA expressions of genes associated with cellular metabolism and mitochondrial biogenesis achieved through endurance exercise (Mahoney et al., 2005). Equally, as resistance training stresses different pathways, maladaptation reported could be resultant of delayed amino acid delivery with blood flow changes (Biolo et al., 1995), or altered macrophage activity and a lesser growth factor concentrations as a result of cold application (Takagi et al., 2011). Regardless, this does point to the need for matching of training stimulus and recovery mechanisms to avoid unfavorable outcomes.

The topic of post-exercise recovery from training has been the focus of recent attention in both narrative (e.g., Nédélec et al., 2013; Minett and Duffield, 2014) and systematic reviews (e.g., Bleakley et al., 2012; Leeder et al., 2012; Bieuzen et al., 2013; Costello et al., 2013; Poppendieck et al., 2013). While these literature detail the physiological, perceptual, and performance effects during recovery, discussion as to the specificity and context within which interventions are best applied is limited. Emphasis should be placed on the matching of the recovery needs (e.g., cellular vs. specific systems, or both) with those affected by any particular recovery approach. As seen with the recent training studies focused on the use of post-exercise cooling recoveries (Fröhlich et al., 2014; Halson et al., 2014), chronic adaptations are affected by recovery choices. In the case of the elite sporting environment where small changes often represent a meaningful difference for performance outcomes, informed decisions surrounding the context of post-exercise recovery (e.g., timing, frequency, exercise mode) are of utmost importance. Areas for future research include consideration for the individual responses to specific recovery methods, influence of athlete preference or perception, and the need to link this to applied practices where sport-specific skill performance, psychology and usability are as valuable as physiological change.

## Conflict of interest statement

The authors declare that the research was conducted in the absence of any commercial or financial relationships that could be construed as a potential conflict of interest.
